# Effects of Cervical Spinal Manipulation on Saccadic Eye Movements

**DOI:** 10.3390/brainsci14030292

**Published:** 2024-03-20

**Authors:** Adam Klotzek, Monem Jemni, Shad James Groves, Frederick Robert Carrick

**Affiliations:** 1 Department of Neurology, Carrick Institute, Cape Canaveral, FL 32920, USA; monemj@hotmail.com (M.J.); shadgroves@hotmail.com (S.J.G.); drfrcarrick@post.harvard.edu (F.R.C.); 2 Centre for Mental Health Research in Association, University of Cambridge, Cambridge CB2 1TN, UK; 3 Faculty of Physical Education, Ningbo University, Ningbo 315000, China; 4 Department of Health Professions Education, MGH Institute of Health Professions, Boston, MA 02129, USA; 5 Burnett School of Biomedical Science, University of Central Florida, Orlando, FL 32827, USA; 6 College of Medicine, University of Central Florida, Orlando, FL 32827, USA

**Keywords:** spinal manipulation, cervical, saccades, concussion, brain, oculography

## Abstract

Quantifying saccadic eye movements can assist in identifying dysfunctional brain networks in both healthy and diseased people. Infrared Oculography is a simple and non-invasive approach to capturing and quantifying saccades, providing information that might aid in diagnosis and outcome assessments. The effect of spinal manipulation on quantified saccadic performance parameters has not been fully studied despite known post-manipulative effects on the brain and brainstem regions controlling them. This case study investigates spinal manipulation’s immediate and long-term effects on saccadic eye movements by quantifying the saccades of a male patient diagnosed with post-concussion syndrome. The patient performed horizontal saccades that were quantified before and immediately following cervical spinal manipulation both at the case study’s start and following a 2-week interim, during which the subject received six manipulative treatments. Immediate and long-term post-manipulative effects were observed, and the results revealed various post-manipulative effects across all quantified parameters in addition to between right and leftward saccades. The immediate post-manipulative effect was greatest at the case study’s onset, while the long-term right and leftward saccadic symmetry were most affected. The observations in this case study demonstrate that cervical spinal manipulation influences saccadic eye movements, providing new insights into its central neurological effects and therapeutic applications beyond its most commonly known use in pain management. More importantly, it encourages scientists to undertake further clinical investigations on wider scales.

## 1. Introduction

Saccadic eye movements are small, rapid eye movements that position the fovea to fixate attention on points of interest for detailed visual processing, thus allowing humans to explore their visual environment quickly [1]. In daily life, they are the most used and effective way of maintaining awareness of the visual environment, which is essential for attention and choice in decision-making. Additionally, they exponentially increase in importance with higher levels of human performance [2].

Historically, the study of eye movements has provided clinicians and researchers with an increasing understanding of brain function in health and disease, beginning in the mid-nineteenth century when the initial accounts of strabismus and nystagmus were reported [3]. More recently, the study of eye movements and their abnormalities has become an established biomarker for many neurodegenerative disorders and a means to evaluate the effect of therapeutic interventions on brain systems [4,5].

Saccades, in particular, have also been studied in various neurological diseases, including post-concussion syndrome (PCS). They act along with other eye movements as a biomarker for the diseases themselves, often revealing impaired persistency throughout a disease’s course. Studying saccadic eye movements also provides clinicians with a better understanding of the neuropathology of the diseases themselves, as compared to normal physiological function [6,7,8,9].

Visual disturbances are common immediately following a concussion. The visual system contains many widely distributed neurological networks that are prone to neurophysiologic changes, resulting in ocular motor dysfunction involving saccadic, smooth pursuit, and vergence eye movements [10,11].

For most individuals who suffer a concussion, symptoms resolve within 2–4 weeks after injury; however, a minority of patients continue to experience persistent symptoms, which appear to be the result of ongoing impairment of ocular motility attributed to ongoing neuronal dysfunction in the brain, biomechanical dysfunction in the cervical spine, and dysfunction in the vestibular system [12,13,14,15].

Infrared oculography is an accurate and non-invasive method of recording saccadic eye movements, and its use and acceptance are growing in clinical practice [16]. It provides both clinicians and researchers with a non-invasive method to accurately assess the integrity of the neural networks involved in producing and controlling saccades, along with objective measurements that are useful in studying the effectiveness of therapeutic clinical trials [17,18].

Cervical spine dysfunction has been known to cause disturbances in saccadic eye movements for quite some time [19,20], with this relationship continuing to be supported by more recent studies investigating impaired saccadic eye movements seen in motor vehicle accident-induced cervical whiplash injuries [21]. It appears that concussion and cervical spine injury occur concurrently. Studies evaluating head impact telemetry reveal that the accelerative force of the head needed to induce a concussion is far greater than that needed to cause damage to soft tissue structures in the neck [14,22]. In a recent review [23], the authors suggested that cervical spine dysfunction, often seen in Whiplash Associated Disorders (WAD), shares common symptomatology and findings as those seen with concussion, and thus, the two may often coexist. Other investigations have also shown a relationship between ocular motility impairment and other non-traumatic causes of cervical spine dysfunction [24,25,26]. The ocular motility impairments seen with cervical spine dysfunction appear to result from impaired proprioceptive input, producing functional alterations in the neural networks controlling various eye movements [21,25,27].

Spinal manipulation can be defined as a therapeutic procedure in which a high-velocity, small-amplitude impulse is applied to a synovial joint of the spine at or near the end of the passive or physiological range of motion [28]. It is often accompanied by an audible “cracking” noise resulting from cavitation of the joint. Spinal manipulation is also most commonly known for its ability to reduce musculoskeletal pain [29], and it has also been shown to improve proprioceptive input and the function of those brain networks involved in producing various eye movements [30,31,32]. However, despite the known effects of altering proprioceptive input into the brain, the effects of cervical spinal manipulation on saccadic eye movements and in patients diagnosed with (PCS), to the best of our knowledge, have not been directly investigated.

This case study investigated whether cervical spinal manipulation has an immediate and or long-term impact on saccadic eye movements by quantifying saccadic latency, amplitude, and velocity parameters immediately following spinal manipulation and a 2-week course of manipulative treatments. We hypothesized that saccadic performance would be affected immediately following spinal manipulation and over an extended period after a series of manipulative treatments. However, we submitted no hypothesis as to the degree to which each parameter would be affected nor its relationship to changes in our patient’s clinical presentation.

## 2. Materials and Methods

### 2.1. Subject

The authors of this case study chose to evaluate a 45-year-old male with PCS due to the prevalence of persistent saccadic eye movement impairments commonly seen with this condition [33,34,35]. The patient was assessed and treated by a board-certified chiropractic neurologist with over 20 years of practice and experience.

The patient reported that he sustained a concussion 15 years prior when he was involved in a collision with another vehicle while on his skateboard. The impact resulted in the patient being thrown onto and shattering the front windshield of the vehicle. The patient was taken by ambulance, on a stretcher with cervical stabilization, to a nearby hospital, where he was evaluated and then released. The impact was severe enough that it took the patient over six months to return to normal physical activity. The patient has never sought any treatment for his post-concussion symptoms and has not had any spinal manipulation performed on his cervical spine. Since the incident, the patient states that he has experienced about a 50% improvement in his post-concussion symptoms. However, he continues to experience decreased ability to focus, decreased mental stamina, global body pain, brain fog, decreased short-term memory, and a decreased ability to learn and retain new information. The patient reported no other documented head injuries.

Written informed consent was obtained for participation, and HIPPA-compliant, anonymized patient information was to be published in this case study.

### 2.2. Saccadic Recording and Quantification

This study utilized an Ober Saccadometer to assess horizontal saccadic performance by capturing and quantifying latency, amplitude, and peak/mean velocity values. Vertical saccades were not assessed in this case study as the Ober Saccadometer is restricted to recording and quantifying horizontal saccadic parameters. For this study’s purpose, we did not see the additional benefit of obtaining vertical saccade measurements.

**Figure 1:**
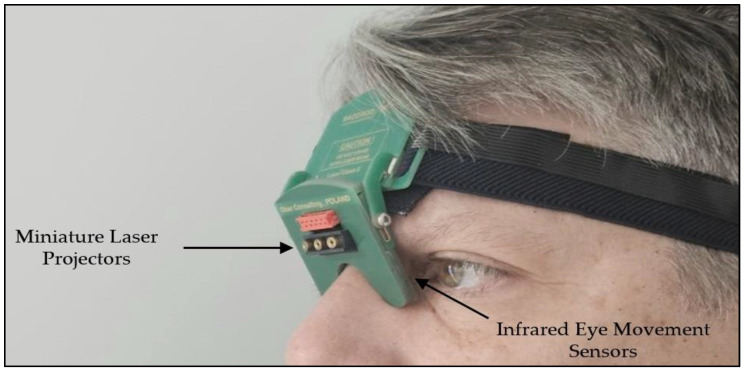
Ober Saccadometer.

The Ober Saccadometer is an infrared oculographic device that performs quantitative evaluations of dynamic saccadic movements, measuring latency, amplitude, and velocity using a micro-miniature device (Figure 1) [36,37]

The saccadometer utilizes direct infrared oculography to measure the left and right eye rotations while performing a horizontal saccade. As a result of the normal conjugation of left and right, horizontal saccadic eye movements can be added and averaged.

The eye position change is calculated by illuminating the inner canthi of the left and right eyes using low-intensity infrared red (IR) light and then measuring the difference between the amounts of IR light reflected from the eye surfaces. The measuring rate utilized by the saccadometer was ±35 degrees with a bandwidth of 1000 HZ, a (peak to peak) noise level of 0.5 arc, and a signal-to-noise ratio of 41.6 dB, using a 10-degree saccade as the reference. The average linearity error utilized was ±15 deg: 1.4 deg, with a maximal accepted error of 2.9 deg.

The infrared oculographic recordings were obtained in the following manner. The subject was seated comfortably 1 m from a wall with uniform texture and a neutral-colored surface to help reduce laser beam scatter hitting the wall. After calibration of the device, data acquisition included the patient performing 100 alternating horizontal saccades (50 to the right and 50 to the left). The patient was instructed to look at an alternating laser dot projected onto the wall as it jumped from right to left. The laser dot targets were projected at a horizontal amplitude of 10° in each direction from the midline, resulting in a total saccade amplitude of 20°. The appearance pace was randomized between 1.3 s and 2.3 s. The device software recorded each eye movement’s position relative to the beginning of the saccade before then calculating the latency (ms), amplitude (deg), peak velocity (deg/s), and mean velocity (deg/s). The recorded data was presented in table and graph format. The graphs are color-coded for the saccade direction (red = left, green = right)

### 2.3. Treatment (Determining the Cervical Segment to Be Manipulated)

The choice of the vertebral segment to manipulate in the cervical spine was determined using the following two assessment criteria. First, we used motion palpation to determine posterior to anterior *z*-axis facet joint translation restriction sidedness. Second, we used muscle testing of the extremities to determine the sidedness of soft pyramidal weakness. We defined the sidedness of soft pyramidal weakness by labeling the side with upper extremity extensor and same-sided lower extremity flexor muscle weakness. The segment chosen was the segment that was restricted in posterior to anterior *z*-axis rotation on the side of soft pyramidal weakness. Based on these two criteria, the C2 cervical segment was chosen.

Each treatment consisted of a single cervical spine manipulation performed similarly by the same licensed Chiropractic Physician holding a Diplomate in Chiropractic Neurology granted by the American Chiropractic Neurology Board [38]. The treating physician was blinded from the results obtained from the saccadic eye recordings, and the eye testing technician was blinded from any information about the patient’s treatment.

**Figure 2:**
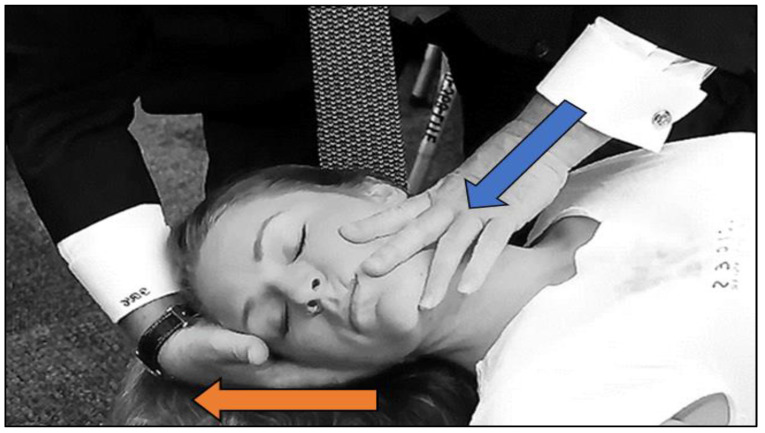
Coupled Cervical Manipulation Force Vectors With Permission [42].

Each cervical spine manipulation was performed utilizing a spinal manipulative technique first described by Carrick [39], with corrective force being applied in a direction to restore normal coupling movement of a pathomechanical moving vertebral segment (Figure 2) [40,41]. Coupled motion in the spine is defined as rotation and translation occurring congruently as the vertebral body moves along one of its cardinal planes. By convention, in a right-handed cartesian coordinate system, “y” represents the vertical axis, “x” represents the horizontal axis, and “z” represents the anteroposterior axis. In the cervical spine, rotation of the vertebral body along the *z*-axis is coupled to rotation around the *y*-axis such that lateral bending to one side (*z*-axis rotation) is coupled to vertebral body rotation to the same side (*y*-axis rotation) [40].

The treating clinician applied a manipulative correction to the C2 cervical segment by placing the manipulative hand on the left side of the cervical spine and producing a thrust to restore the −theta *z*-axis rotation coupled to the +theta *y*-axis rotation to the C2 vertebral segment (Blue Arrow). The right hand was placed on the right side of the neck, applying a traction force along the *y*-axis (Orange Arrow: Figure 2).

**Figure 3:**
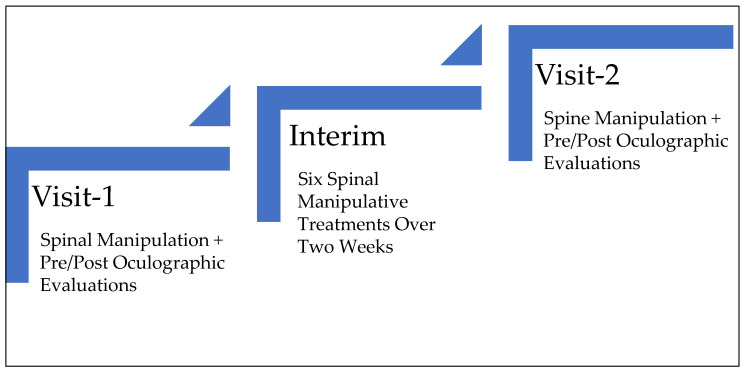
Evaluation and Treatment Outline.

The saccades performed in the case study were quantified before and immediately following cervical spinal manipulation both at the case study’s start (Visit-1) and following a 2-week interim (Visit-2), during which the subject received six cervical spinal manipulative treatments (Interim), as outlined in (Figure 3).

The mean saccadic test parameter values (latency, amplitude, peak/mean velocity) were used to calculate the percentage difference between the means, which was expressed as either a negative percentage (decreased) or a positive percentage (increased), depending on how the means were compared. For instance, if we compared the post-mean value to the pre-mean value and that comparison resulted in the post-value being less than the pre-value, the percentage change would be expressed as a negative change.

Mean Saccadic Performance Parameter Value Comparisons Performed:

1. *Immediate Treatment Effect:* Separately compares the post to pre-treatment saccadic parameter values in visits 1 and 2.
2. *Long-Term Treatment Effect:* Compares the pre-treatment saccadic parameter values of visit 2 to 1.
3. *Immediate Treatment Effect Bias:* Compares the post-treatment saccadic parameter values in visits 1 and 2.
4. *Right and Left Asymmetry Effect*: Compares the saccadic parameter values between right and leftward saccades.

## 3. Results

### 3.1. Visit-1

#### 3.1.1. Visit-1: Immediate Treatment Effect

**Figure 4:**
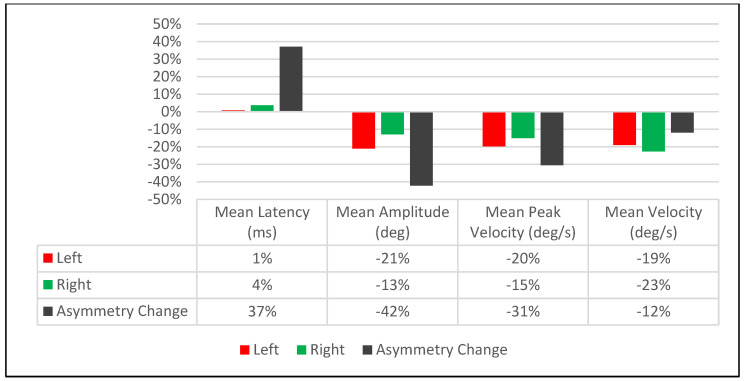
Visit-1: Immediate Treatment Effect.

The post-treatment mean amplitude and saccadic velocity values decreased for both right and leftward saccades while the mean latency values increased. The post-treatment effect was non-uniform across all parameters and asymmetric regarding the degree of effect on right and leftward saccades. For instance, saccadic mean amplitude values decreased by 21% for rightward saccades and 13% for leftward saccades post-treatment (Table 1), resulting in an overall decrease in amplitude asymmetry between right and leftward saccades by 42% (Table 1). However, saccadic latency values increased by a lower margin than the amplitude values (1% for rightward saccades and 4% for leftward saccades), resulting in an overall increase in latency asymmetry between right and leftward saccades by 37% (Table 1 and Figure 4).

#### 3.1.2. Visit-1 Saccadic Parameter Profiles: Immediate Treatment Effect

**Figure 5:**
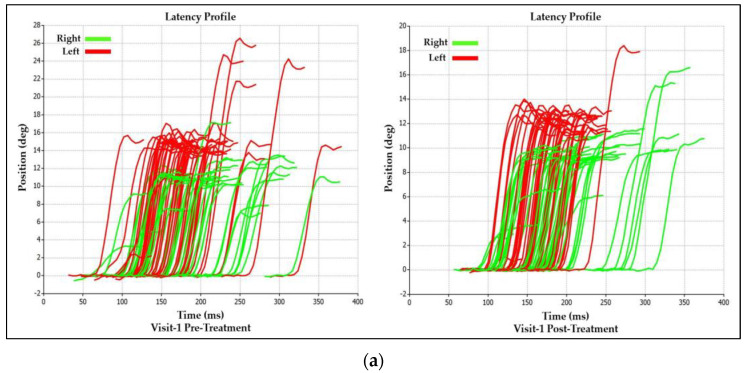

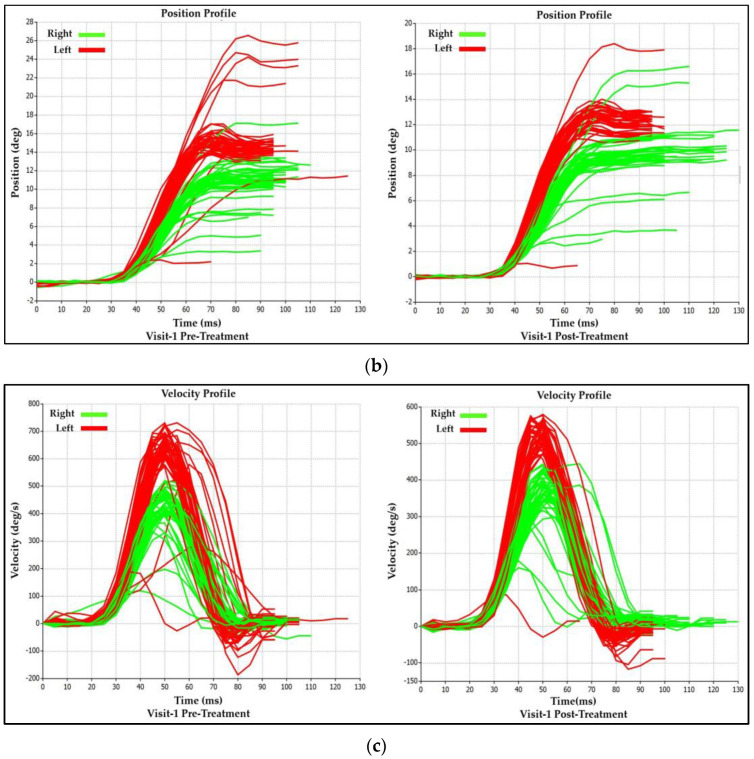
(**a**) Visit-1 Latency Profiles: Immediate Treatment Effect. (**b**) Visit-1 Position (Amplitude) Profiles: Immediate Treatment Effect. (**c**) Visit-1 Velocity Profiles: Immediate Treatment Effect.

The immediate treatment effect of Visit-1 can also be demonstrated by observing the post-treatment changes in the saccadic profile tracings (Figure 5a–c), which coincide with the analytical data presented in Table 1. It is visible that the increase in post-treatment latency asymmetry appears secondary to the rightward shift of the rightward saccade tracings (Green) as compared to the leftward (Red) saccade tracings in the post-treatment profile (Figure 5a). The decrease in amplitude and velocity asymmetry between right and leftward saccades post-treatment is easily observed by the greater degree of overlap between rightward (Green) and leftward (Red) saccade tracings in the post-treatment position (amplitude) and velocity profiles, as well as the decreased number of outliers that deviate from the general clustering, which is a visual representation of the mean values (Figure 5b,c).

### 3.2. Visit-2

#### 3.2.1. Visit-2: Immediate Treatment Effect

**Figure 6:**
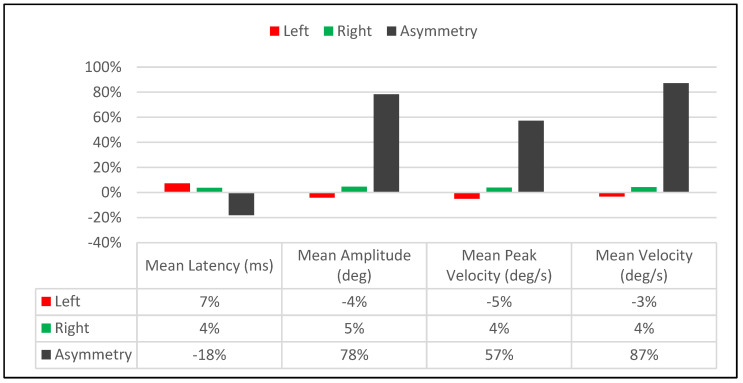
Visit-2: Immediate Treatment Effect.

The immediate treatment effect of Visit-2 was significantly different than Visit-1. Whereas in Visit-1, there was an observed reduction in the asymmetry between right and leftward saccades involving amplitude and velocity (Table 1, Figure 4)**,** Visit-2 did the opposite by increasing the asymmetry between right and leftward saccades for those parameters (Table 2, Figure 6). For saccadic latency values, Visit-2 increased the right and leftward saccade values by 4% and 7%, respectively, while decreasing the latency asymmetry between them by 18% (Table 2, Figure 6). This was in contrast to the post-treatment effects of Visit-1, which saw the latency asymmetry between right and leftward saccades increase by 37% (Table 1, Figure 4).

#### 3.2.2. Visit-2 Saccadic Parameter Profiles: Immediate Treatment Effect

**Figure 7:**
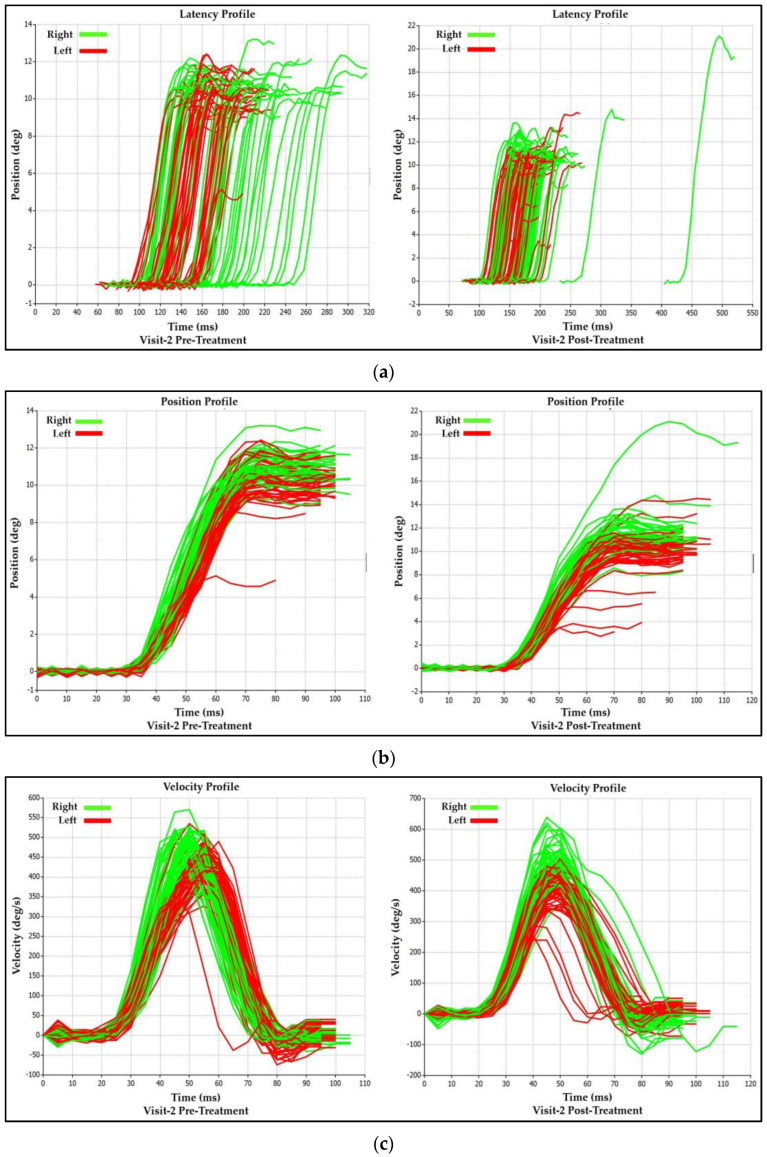
(**a**) Visit-2 Latency Profiles: Immediate Treatment Effect. (**b**) Visit-2 Position (Amplitude) Profiles: Immediate Treatment Effect. (**c**) Visit-2 Velocity Profiles: Immediate Treatment Effect.

As in Visit-1, the acute treatment effect of Visit-2 can also be demonstrated by observing the changes in the saccadic profile tracings (Figure 7a–c), which coincide with the analytical data presented in Table 2. The observed post-treatment decrease in rightward (Green) and leftward (Red) saccadic asymmetry is visible by a leftward shift of the rightward (Green) saccade latency tracings in the post-treatment graphs. This shift creates a more significant overlap between the leftward (Red) saccade latency tracings signifying greater symmetry (Figure 7a Increases in the asymmetry between right and leftward saccadic amplitude and velocity values can be observed through a decrease in the degree of overlap in the rightward (Green) and leftward (Red) saccade tracings in the position (amplitude) and velocity plots when comparing post- treatment to pre-treatment plots (Figure 7a,b).

### 3.3. Long-Term Treatment Effect

**Figure 8:**
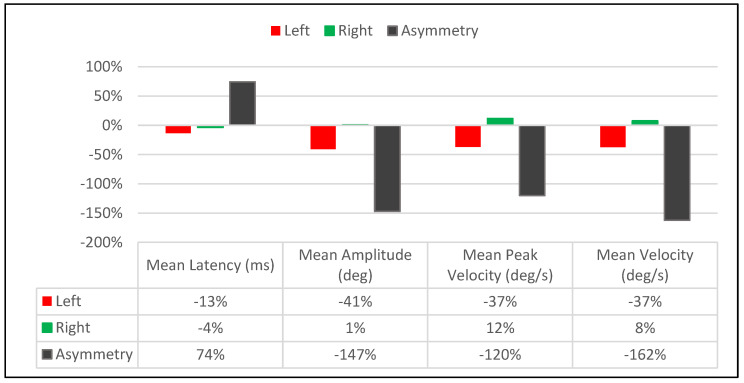
Long-Term Treatment Effect: Visit-1 and 2 Comparison.

The long-term treatment effect resulted in non-uniform and asymmetrical value changes between the right and leftward saccades across all performance parameters consistent with what was observed in the immediate post-treatment effect in Visits 1 & 2. However, the non-uniform changes in saccadic amplitude and velocity values and the asymmetrical changes between the right and leftward saccades resulted in a considerable reduction in asymmetry in those values between the right and leftward saccades. In contrast, the latency asymmetry values between the right and leftwards saccades increased (Figure 8).

#### Saccadic Parameter Profiles: Long-Term Treatment Effect

**Figure 9:**
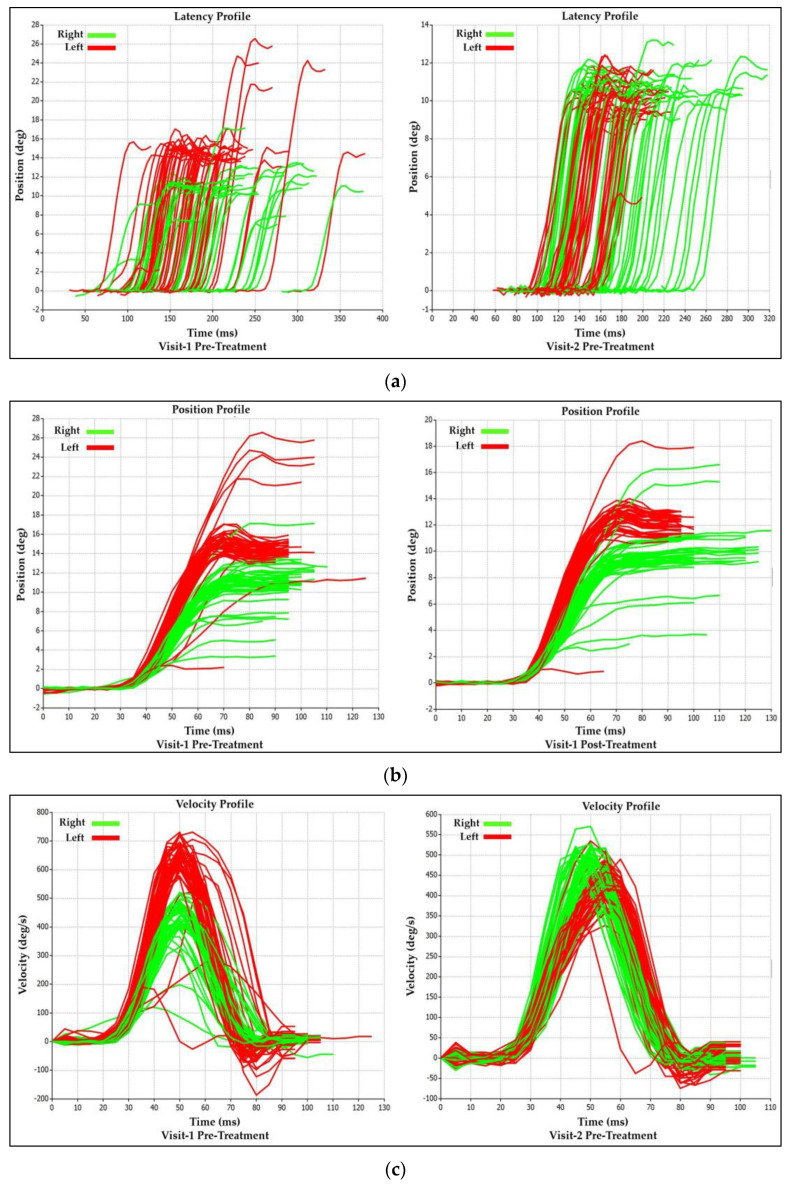
(**a**) Latency Profiles: Long-Term Treatment Effect. (**b**) Position (Amplitude) Profiles: Long-Term Treatment Effect. (**c**) Velocity Profiles: Long-Term Treatment Effect.

The saccadic performance profile tracings provide an excellent visual representation of the long-term treatment effect observed in the analytical data in (Figure 9a–c). The latency profiles between pre-treatment values from Visit-1 to Visit-2 show an overall shift to the right for rightward (Green) saccade tracings as compared to the leftward (Red) saccade latency tracings (Figure 9a), coinciding with the 74% increase asymmetry between right and leftward saccadic latency values from Visit-1 to Visit-2 (Figure 8). The position (amplitude) and velocity profiles show an increased overlap of the rightward (Green) and Leftward (Red) saccade tracings from Visit-1 to Visit-2. The increased overlap (Improved Symmetry) was a result of an upward shift of the leftward (Red) saccade tracings and a downward shift of the rightward (Green) saccade tracings. This coincides with a decrease in amplitude and velocity asymmetry between the right and leftwards saccades from Visit-1 to Visit-2 (Figure 8 and Figure 9b,c).

### 3.4. Immediate Treatment Effect Bias between Visit-1 and 2

**Figure 10:**
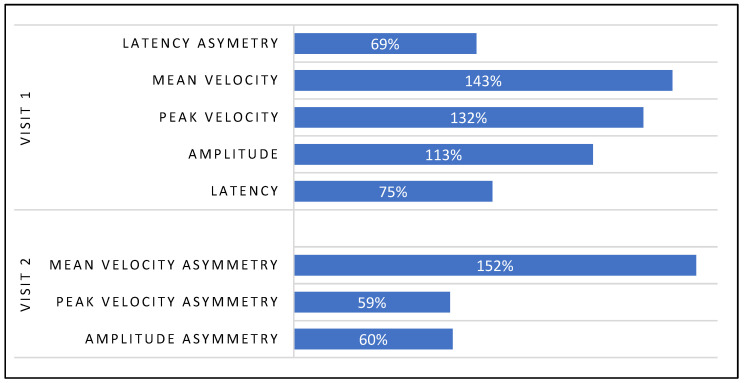
Immediate Treatment Effect Bias Between Visit-1 and 2.

The immediate treatment effect observed in Visit-1 was significantly greater than in Visit-2. Visit-1 had a greater effect on more performance parameters than Visit-2, and, on average, Visit-1 had a greater percentage effect over Visit-2 (Figure 10).

## 4. Discussion

### 4.1. Investigative Purpose

This case study investigated whether cervical spinal manipulation affects horizontal saccadic eye movements by observing the immediate and long-term post-manipulative effects on saccadic latency, amplitude, and velocity parameters in an individual patient with PCS.

### 4.2. Primary Outcomes

Three primary outcomes were observed in this case study. First, the effect of cervical spinal manipulation, both immediate and long-term, varied for right and leftward saccades across all quantified parameters in degree, uniformity, and each manipulative event. Second, the immediate post-manipulative effect differed between the initial and last visits, such that a greater overall effect was observed on the initial visit. Third, the overall long-term manipulative effect was a large decrease in asymmetry between right and leftward saccades for amplitude and velocity parameters, while latency asymmetry increased.

### 4.3. Choice of Saccadic Recording Device and Protocol

The field of saccadic eye movement quantification has been evolving, with new technologies being introduced to address the lack of user-friendliness and complexity inherent in older systems [43]. Contemporary studies on saccadic eye movements vary widely regarding utilized protocols and data analysis, often being dependent on the investigators’ device-specific software [17,44,45,46].

We chose our protocol and the Ober Saccadometer recording device due to its simplicity, accuracy, and ability to obtain saccadic recordings more representative of what happens in a clinical setting [47].

### 4.4. Choice of Horizontal Saccade Evaluation over Vertical Saccades

We chose to evaluate only horizontal saccades in this case study due to the restrictions of the Ober Saccadometer’s recording ability in regard to horizontal saccades. Some may argue that this case study should have included vertical saccadic measurements based on evidence suggesting that certain neurological disorders, traumas, and age have differing degrees of effects on horizontal and vertical saccadic eye movements [48,49,50,51]. We did not feel that distinguishing between vertical and horizontal saccade effects was pertinent to this case study’s investigative purpose, which was to demonstrate that cervical spinal manipulation impacts saccadic performance parameters. Determining whether that impact differs for vertical or horizontal saccades, disease state, and age are questions to be answered in future investigations.

### 4.5. Saccadic Eye Movements: A Biomarker of Neurological Disease

The quantification of saccadic eye movements provides clinicians with a simple, accurate, and cost-effective way to ascertain the activity of various brain networks both in health and disease. This can provide clinicians with valuable insights into the effects of various disease processes on multiple brain networks and information as to the impact of a particular therapeutic intervention on them [52,53]. Impairments in saccadic eye movements have been associated with a higher symptom burden and reduced white matter integrity in patients with post-concussion syndrome [54], as well as in sports-related concussions [10]. Slowing of saccadic eye movements is commonly seen in cerebellar disease, progressive supranuclear palsy, and spinocerebellar ataxia type 2 [55]. The measurement of saccadic eye movements has also been shown as an effective biomarker for the diagnosis, progression, and degree of cognitive impairment seen in Parkinson’s disease [56].

### 4.6. Structural and Functional Brain Asymmetry

Structural and functional brain asymmetry appears to be a commonality as opposed to a rarity in vertebrates and humans, though it varies more in humans than in primates [57]. In a recent review, the authors concluded that a selective leftward dominance for language accompanies a general right hemispheric dominance [58]. However, task demands may influence this general relationship, with different tasks temporarily activating the hemispheric suitable loops of feedforward or feedback projections dominating a neural process [59]. Hemispheric functional asymmetry is also influenced by environmental stressors that increase cortisol levels, resulting in a higher relative left frontal activity, particularly in individuals with low in-action orientation [60]. Time also affects hemispheric lateralization in that it is not fixed and changes in strength over an individual’s lifespan, as research conducted on chicks has shown that visual lateralization changes markedly over early and critical stages of development and can be modulated by steroid hormones and environmental stimulation [61].

### 4.7. Saccadic Generation and Visual System Asymmetry

The saccadic generation and control centers are widely distributed among various brain, brainstem, and cerebellar regions [1,62,63,64]. As previously described, functional hemispheric asymmetry also influences saccadic eye movements, as saccadic accuracy appears sensitive to functional hemispheric asymmetries, such as lateralizing visuospatial attention in the right hemisphere [65].

Asymmetries in the visual system have also been shown to influence saccadic peak velocity and latency values [66]. Genetic polymorphisms affecting the brain also appear to influence saccadic eye movements. Researchers examining the effects of the catecholamine-O-methyltransferase (COMT) gene Val158Met polymorphism on antisaccade performance found asymmetrical effects depending on the inherited polymorphism. Carriers of met/met homozygotes had more significant errors in producing antisaccades in response to leftward stimuli than right. In contrast, for carriers of the Val allele, the saccadic latency for the left stimuli was significantly higher than that for the right. The authors concluded that there was a relative decrease in the efficiency of the right hemisphere in met/met genotype carriers and the left hemisphere in the Val/Val genotype [67].

### 4.8. Neurophysiological Effects of Spinal Manipulation

Spinal manipulation has multiple effects on the peripheral and central nervous systems [29,31,68], which are thought to occur secondary to alterations in post-manipulative proprioceptive input [69,70,71].

Cervical spine proprioception has been shown to influence oculomotor control [72], and impairment can produce sensorimotor disruption, affecting visual functions influenced by the cervical spine [73,74]. Treatments focusing on improving proprioceptive input from the cervical spine and improving head−eye coordinative movements have been observed to improve saccadic performance [75,76]. Spinal manipulation also improves proprioception, as observed through a post-manipulative reduction in Joint Position Error (JPE) values [77,78,79]. It has been suggested that the post-manipulative effect on saccadic movements is most likely due to improved proprioceptive input from the cervical spine [30].

The effects of spinal manipulation on the neural networks of the brain appear to differ significantly between the right and left hemispheres, as observed by the post-manipulative effects on neural network structure and connectivity [80]. In examining the effects of lumbar spinal manipulation on chronic low back pain patients using Bold fMRI, authors found asymmetrical hemispheric post-treatment changes in Reho values. The authors observed a left hemispheric bias affecting the left precuneus, superior frontal, and postcentral posterior cingulate gyri asymmetrically [81]. Another similar study measuring the fMRI to measure the brain effects of lumbar spinal manipulation on individuals with chronic low back pain showed asymmetrical increases in hemispheric activity positively correlating to lower pain scores [82], while authors examining the brain effects of lumbar spine manipulation on individuals with disc herniation using fMRI found asymmetrical modulation of suspected dysfunctional brain regions essential for pain processing [83]. In a study examining the effects of spinal manipulation on the N30 somatosensory evoked potential, authors found a 31.3% decrease in the amplitude of the N30 potential within the prefrontal cortex after spinal manipulation, suggesting an alteration of somatosensory processing post-manipulation. However, the authors did not test for right or left hemispheric differences [32]. Differing right and left hemispheric effects also appear to be influenced by which side of the spine the manipulative thrust occurs on [84], by evidence suggesting asymmetrical brain effects from unilateral extremity joint manipulation [85] and electroacupuncture application [86].

Spinal manipulation has also been observed to alter local spinal reflexes and muscle tone. The degree of effect appears to depend on the magnitude and speed of the thrust utilized [87,88]. These effects appear asymmetrical and complex, adding to the variability observed in studies measuring spinal manipulative effects on functional brain activity [89,90,91].

### 4.9. Horizontal Gaze Shifts and Neck Musculature Recruitment

Horizontal gaze shifts from right to left sides have been observed to asymmetrically recruit neck musculature to facilitate head movement in the direction of gaze. Using transcranial magnetic stimulation (TMS), authors have observed that the application of TMS to the frontal eye field (FEF) decreased contralateral reaction times and activated neck musculature in a pattern consistent with the recruitment of a contralateral head-turning synergy [92]. Stimulation of the Superior Colliculus in primates to produce contralateral saccades has been shown to evoke EMG activity predominantly in the contralateral obliquus capitis inferior, rectus capitis posterior major, and splenius capitis muscles. The coupling of saccadic eye movements and neck musculature recruitment results in synergistic head-turning in the direction of a saccade [93], suggesting that impairments in saccadic eye movement would also promote biomechanical dysfunction in the cervical spine.

### 4.10. Case Study’s Perspective

The limited studies investigating the effects of spinal manipulation on brain activity have yielded inconsistent observations regarding affected brain regions within and between each hemisphere. The inconsistent observations are to be expected given the brain’s normal structural and functional asymmetry resulting from the integrated interactions of genetic and environmental factors that influence neuronal development and connectivity in utero and throughout one’s life [94,95,96].

The lack of structural and functional symmetry within and between the brain’s hemispheres presents a significant challenge for clinicians and researchers investigating the effects of a particular treatment on the brain. The challenge becomes accentuated when investigating the effects of spinal manipulation, as the post-manipulative effects on proprioceptive input are inherently asymmetrical, occurring because of the different vectors of force being applied to the right and left sides of the spine during the manipulative event (Figure 2).

An interesting and important observation in this case study was that, despite the non-uniform and asymmetrical immediate post-manipulative effects, the long-term effect reduced the overall saccadic asymmetry for amplitude and velocity between the right and leftward saccades, suggesting improved neural network synchronization between hemispheres for those parameters. In contrast, the opposite occurred with saccadic latency, as latency asymmetry increased long-term between the right and leftward saccades.

Considering the brain’s normal structural and functional asymmetry, combined with the asymmetrical post-manipulative effect on proprioceptive input, clinicians must consider these factors when predicting or interpreting the central neurological effects of spinal manipulation. Clinicians and researchers may also want to consider the cumulative effects of the prior manipulations, the reproducibility of the manipulative thrust, and the patient’s attentiveness as factors influencing the post-manipulative effect [97,98].

Lastly, numerous studies are currently looking at the holistic treatments of neuro-diseases and mental health issues from different angles and by associating a few assessments. For instance, Jemni et al. [99] have cited brain-derived neurotrophic factor (BDNF) as a biomarker while introducing exercise as a non-medicinal alternative to treat depression. We are therefore intrigued about associating cervical manipulation with other invasive biomarkers for concussion. Such studies could provide further insights into how the brain and the body react to short- and long-term stimuli.

## 5. Conclusions

This case study investigated cervical spinal manipulation’s immediate and long-term effects on saccadic eye movements by observing the post-manipulative changes in saccadic latency, amplitude, and velocity parameters.

This case study demonstrates that cervical spinal manipulation has an immediate and long-term effect on saccadic eye movements. The post-manipulative effect varied in degree, uniformity, and dates performed, and it was asymmetrical between right and leftward saccades across all the saccadic performance parameters measured. The non-uniform and asymmetrical post-manipulative effects observed in this case study suggest that quantifying saccadic eye movements using Infrared Oculography can provide clinicians and researchers with vital information regarding spinal manipulation’s central neurological effects.

In summary, the findings of this case study suggest that spinal manipulation may have broader therapeutic uses other than in the field of pain management, for which it is most commonly known. Given that impaired saccadic eye movements are associated with many brain-based neurological disorders, seeing a clear post-manipulative effect suggests that it may also alter the patient’s symptomatology and the general clinical presentation of the patient.

## 6. Limitations and Recommendations

### 6.1. Limitations

The findings observed in this case study may not apply to the general population. Since the data were collected on a single patient, meaning statistical certainty cannot be assessed, it is recommended that larger studies be undertaken to check the probabilities and the cause to effect. It is recommended that further clinical investigation be performed to validate the findings observed in this case study. This case study only tested horizontal saccades; the observed findings may not extrapolate to vertical saccades or other eye movements. In addition, the head of the patient had not been stabilized when measuring the saccades, which may have affected the reliability of the test and retest.

### 6.2. Recommendations

The findings of this case study suggest that cervical spinal manipulation affects the neural networks modulating saccadic eye movements, thus affecting various saccadic performance parameters. Given that saccadic eye movements are often impaired in many neurological disorders, accurately analyzing them can provide valuable information as to a subject’s neurological condition and how that condition may respond to treatment [100]. It also allows for the investigation of possible new therapeutic uses of a treatment modality not commonly used for a specific condition.

Demonstrating that spinal manipulation can alter saccadic eye movements raises the question of its possible use in treating conditions other than pain, for which it is most commonly known. In light of these findings, we recommend that researchers further investigate the therapeutic role of spinal manipulation outside of the context of pain and that clinicians consider its use in patients with impaired saccadic eye movements, especially if other therapeutic interventions have failed to yield positive results. Larger clinical studies within this particular context would be greatly beneficial.

## Figures and Tables

**Table 1:** Visit-1: Immediate Treatment Effect.

Saccadic Performance Parameter	Left Saccade	Right Saccade	R/L Asymmetry
Mean Latency (ms)			
*Pre-Treatment*	151	162	11
*Post-Treatment*	152	168	16
*Delta*	1	6	5
*% Change*	1%	4%	37%
Mean Amplitude (deg)			
*Pre-Treatment*	15.3	10.7	4.6
*Post-Treatment*	12.4	9.4	3
*Delta*	2.9	1.3	1.6
*% Change*	−21%	−13%	−42%
Mean Peak Velocity (deg/s)			
*Pre-Treatment*	630	430	200
*Post-Treatment*	517	370	147
*Delta*	113	60	53
*% Change*	−20%	−15%	−31%
Mean Velocity (deg/s)			
*Pre-Treatment*	317	211	106
*Post-Treatment*	262	168	94
*Delta*	55	43	12
*% Change*	−19%	−23%	−12%

**Table 2:** Visit-2: Immediate Treatment Effect.

Saccadic Performance Parameter	Left Saccade	Right Saccade	R/L Asymmetry
Mean Latency (ms)			
*Pre-Treatment*	132	156	24
*Post-Treatment*	142	162	20
*Delta*	10	6	4
*% Change*	7%	4%	−18%
Mean Amplitude (deg)			
*Pre-Treatment*	10.1	10.8	0.7
*Post-Treatment*	9.7	11.3	1.6
*Delta*	0.4	0.5	0.9
*% Change*	−4%	5%	78%
Mean Peak Velocity (deg/s)			
*Pre-Treatment*	433	483	50
*Post-Treatment*	412	502	90
*Delta*	21	19	40
*% Change*	−5%	4%	57%
Mean Velocity (deg/s)			
*Pre-Treatment*	217	228	11
*Post-Treatment*	210	238	28
*Delta*	7	10	17
*% Change*	−3%	4%	87%

## Data Availability

The original contributions presented in the study are included in the article. Further inquiries can be directed to the corresponding authors.
